# The Microbiological Background of Medication-Related Osteonecrosis of the Jaw (MRONJ): Clinical Evidence Based on Traditional Culture and Molecular Biological Detection Methods

**DOI:** 10.3390/antibiotics14020203

**Published:** 2025-02-15

**Authors:** Zsanett Kövér, Márió Gajdács, Beáta Polgár, Dóra Szabó, Edit Urbán

**Affiliations:** 1 Department of Dentistry, Oral and Maxillofacial Surgery, Medical School, University of Pécs, Tüzér u. 1., 7623 Pécs, Hungary; kover.zsanett@pte.hu; 2 Department of Oral Biology and Experimental Dental Research, Faculty of Dentistry, University of Szeged, Tisza Lajos krt. 64-66., 6725 Szeged, Hungary; gajdacs.mario@stoma.szote.u-szeged.hu; 3 Department of Medical Microbiology and Immunology, Clinical Center, University of Pécs, Szigeti út 12., 7624 Pécs, Hungary; polgar.beata@pte.hu; 4 Institute of Medical Microbiology, Semmelweis University, Nagyvárad tér 4., 1089 Budapest, Hungary; szabo.dora@med.semmelweis-univ.hu; 5 Department of Neurosurgery and Neurointervention, Semmelweis University, Amerikai út 57., 1085 Budapest, Hungary; 6 HUN-REN-SU Human Microbiota Research Group, 1052 Budapest, Hungary

**Keywords:** medication-related osteonecrosis, MRONJ, oral microbiota, *Actinomyces* spp., anaerobic bacteria, culture, 16S rRNA sequencing

## Abstract

**Background**: Medication-related osteonecrosis of the jaw (MRONJ) is a common adverse event following antiresorptive treatment, leading to chronic inflammation and exposed, necrotic bone surfaces in the jawbone. There is an increasing recognition of the role of compositional changes in the colonizing members of the oral microbiota implicated in triggering and/or maintaining MRONJ. The aim of our study was to characterize the culturable and non-culturable microbiota—with particular focus on *Actinomyces* spp. and *Actinomyces*-like organisms (ALOs)—from surgically removed bone samples of MRONJ patients and healthy control subjects. **Methods**: *n* = 35 patients (median age: 70 years) in various stages of MRONJ, with a history of receiving oral or intravenous antiresorptive treatment were included in the study. The controls (*n* = 35; median age: 35 years) consisted of otherwise healthy individuals undergoing tooth extraction. Traditional, quantitative, aerobic, and anaerobic culture, and *Actinomyces*-specific PCR was performed for all bone samples from patients and controls, while microbiome analyses—based on 16S rRNA sequencing—were carried out in 5-5 randomly selected samples. Mann–Whitney U test, Wilcoxon rank sum test (alpha diversity), and PERMANOVA analysis (beta diversity) were performed. **Results**: In MRONJ samples, 185 anaerobic isolates, corresponding to 65 different species were identified (vs. 72 isolates, corresponding to 27 different species in the control group). The detection of *Actinomyces* spp. and ALOs was more common in MRONJ bone samples, based on traditional culture (65.7% vs. 17.1%; *p* < 0.001) and PCR (82.9% vs. 37.1%; *p* < 0.001), respectively. The isolation of *Fusobacterium* spp. (22 vs. 7; *p* = 0.001), *Prevotella* spp. (22 vs. 6; *p* = 0.034), and Gram-positive anaerobic cocci (GPAC) (30 vs. 9; *p* = 0.016) was significantly more common in MRONJ patient samples. The microbiota of the controls’ bone samples were characterized by a considerable dominance of *Streptococcus* spp. and *Veillonella* spp, while the bacterial abundance rates were substantially more heterogeneous in MRONJ bone samples. Notable differences were not observed among the samples related to the abundance of *Actinomyces* in the bone microbiota. **Conclusions**: According to the “infection hypothesis”, alterations in the oral microbiome—with *Actinomyces* and ALOs being the most relevant—may play a key role in the development, aggravation, and progression of MRONJ. The timely detection of *Actinomyces* in necrotic bone is crucial, as it has important therapeutic implications.

## 1. Introduction

Medication-related osteonecrosis of the jaw (MRONJ)—a clinical entity that was previously termed as bisphosphonate-related osteonecrosis of the jaw (BRONJ)—is characterized by a denuded surface area or a lesion of the jawbones, which is accessible through an intra- or extra-oral fistula in the maxillofacial region, persisting for ≥8 weeks after its initial identification by a healthcare professional, in patients with a past medical history of receiving specific medications, but without radiotherapy or malignancy of the jawbones [1,2,3]. The first report of MRONJ was by Marx et al. in 2003 [4], termed as “induced avascular necrosis”, where the case series of 36 patients were described with symptoms of severe jaw pain, denuded bone surfaces, and the formation of abscesses and osteomyelitis, all of whom had received pamidronate or zoledronate treatment. Bisphosphonates (PPs) were introduced into clinical practice in the late 1960s (i.e., etidronate) and are used in numerous contexts, including osteoporosis-related (fragility) fractures, Paget’s disease of bone, and myeloma multiplex (MM), and also in cancers, where bone metastases are common (e.g., breast, prostate, lung, kidney, thyroid) [5,6]. The administration of PPs was an effective means for improving or maintaining the quality of life (QoL) of affected patients (associated with the decreased incidence of pathological fractures, an inhibition or slow-down of bone metastasis formation, less bone pain, and the better management of hypercalcemia), which has led to their widespread use [7,8]. Nevertheless, with their increasingly common use—and with the emergence of combined and sequential therapies—there was a steep rise in the frequency of reported adverse events (AE) associated with these treatments [9]. One of the more severe AEs of PPs is osteonecrosis, which majorly affects bones with high osteoclast activity and remodeling rates, which may explain why the jawbones are most commonly impacted [10].

Although osteonecrosis of the jaw was mostly associated with bisphosphonate treatment (BRONJ), the American Association of Oral and Maxillofacial Surgeons (AAOMS) recommended the revision of the terminology to MRONJ in 2014 (which was followed up by an additional revision in 2022) [11,12]. On the one hand, osteonecrosis of the jaw was described in patients who did not receive PPs (non-BRONJ), but other antiresorptive treatments (e.g., receptor activator of nuclear factor kappa beta-ligand [RANK-L] inhibitor denosumab), and drugs of other pharmacological varieties (e.g., anti-vascular endothelial growth factor [anti-VEGF] monoclonal antibodies, such as bevacizumab, ramucirumab; interleukin-6 receptor inhibitors, such as tocilizumab; tyrosine kinase-based angiogenesis inhibitors, such as sunitinib, pazopanib, sorafenib; and mammalian target of rapamycin [mTOR] inhibitors, such as everolimus) [13,14]. Furthermore, antiresorptive medications with improved pharmacokinetic properties, higher potency, and the use of antiresorptive combinations have also increased the frequency and burden of MRONJ [15,16]. Patients may be classified as low- or high-risk for developing MRONJ and other AEs, based on various non-modifiable characteristics (sex, advanced age), the patients’ underlying conditions and co-morbidities (i.e., diabetes [DM], kidney disease, hypertension, immune suppression), lifestyle factors (e.g., tobacco use (T), alcohol consumption (A), inadequate oral hygiene practices), the type, dose, duration, and administration route of antiresoptive medications (e.g., intravenous administration of PPs is associated with 100–1000-times higher risk of developing MRONJ vs. their use *per os*), and the concurrent use of other treatment modalities (e.g., hormone therapy, steroid use, radiotherapy) [17,18,19,20]. Additionally, specific single nucleotide polymorphisms (SNPs) in genes encoding farnesyl diphosphate synthase (*FDPS*), Sirtuin-1 (*SIRT1*, a specific group of histone deacetylase proteins), and cytochrome P450 2C8 (*CYP2C8*) were described as having heightened risk for developing MRONJ [21,22].

Based on a retrospective, claims-based analysis of the National Health Insurance Fund (NHIF) for 2010–2014, ~0.5% of the Hungarian population has undergone PP treatment [23]. According to previous studies, the prevalence of MRONJ was shown to range between 0.02 and 3% among patients with osteoporosis, while this was between 0 and 18% in patients with cancer, respectively [24,25,26,27]; based on the data of the NHIF, the prevalence of MRONJ in Hungary was 0.1% and 0.9% for individuals without and with a malignancy, respectively [23].

The current understanding of the pathogenesis of MRONJ is that the illness is multifactorial in nature, with the previously described determinants acting as risk factors [28]; however, there is no evidence available to comprehensively explain the pathway to the development of necrotic bone lesions and tissue destruction. PP treatment clearly plays a role, as it impairs the bone’s remodeling capacity and blood supply and causes corresponding immune dysfunction [29]. This escalates the risk of developing osteomyelitis and jaw osteonecrosis via exogenous insults following mucosal damage (e.g., due to trauma), often leaving limited therapeutic options, other than surgical resection, in the arsenal of clinicians [30]. Furthermore, several recent studies suggest that PPs may directly affect immune function by compromising the activity of immune cells and their interactions with bone tissue [31]. On the other hand, there is an increasing recognition of the role of qualitative and quantitative compositional changes in the colonizing members of the oral microbiota, often implicated in triggering and/or maintaining MRONJ [32].

The oral microbiota contains at least >700 species of microogranisms, predominantly consisting of anaerobic bacteria [33]. Among them, there is increasing number of reports highlighting role of *Actinomyces* spp. and *Actinomyces*-like organisms (ALOs, e.g., *Actinotignum* spp., *Arcanobacterium* spp., *Trueperella* spp.) in the development and exacerbation of MRONJ and other types of jaw osteonecrosis [34,35]. *Actinomyces* spp. and ALOs are anaerobic, non-spore-forming Gram-positive rods—abundantly found in the oral microbiota under physiological conditions—characterized by low virulence and a lack of tissue degrading enzymes; therefore, they are unable to penetrate an intact mucosa, but may commonly be found in infections (actinomycosis) where anatomical barriers are breached, leading to chronic illnesses [36]. The genus *Actinomyces* has been subject to substantial taxonomic reclassification, where numerous clinically relevant species received new taxonomic designations (such as *Schaalia odontolytica* from *A. odontolyticus*, *Winkia neuii* from *A. neuii*, *Gleimella europaeus* from *A. europaeus*, *Pauljensenia hongkongensis* from *A. hongkongensis*, and *Bowdeniella nasicola* from *A. nasicola*), resulting in a more heterogeneous group of pathogens [37]. Additionally, the detection of *Actinomyces* in necrotic jaw tissue was associated with longer recovery times [38,39]. Nevertheless, the association between the presence of *Actinomyces* spp. and ALOs and MRONJ is still poorly understood, i.e., it is unknown whether the primary cause of MRONJ is due to antiresorptive treatment (and associated pathophysiological changes involving bone remodeling and immune dysfunction), with the presence of *Actinomyces* strains being secondary, with these strains only acting as colonizers in the necrotic bone, or whether PP therapy is just a precondition to provide access for *Actinomyces* spp. and ALOs to cause inflammation and manifest osteomyelitis and subsequent osteonecrosis [40].

Due to the aging of the population and the corresponding morbidity patterns, it is not reasonable to assume that the number of patients receiving antiresorptive treatments is expected to rise in the future [41]. As MRONJ is one of the major AEs associated with the use of these drugs, the disease burden of this condition may also notably rise; as MRONJ considerably influences functionality and the QoL of affected patients, it is imperative to explore and identify factors that may trigger, maintain, or worsen the disease, to provide effective preventive and therapeutic interventions [42]. Thus, our comparative study was aimed to characterize the culturable and non-culturable microbiota—with particular focus on *Actinomyces* spp. and ALOs—from surgically removed bone samples taken from MRONJ patients receiving antiresorptive treatment and healthy control subjects at a tertiary-care hospital in the Southern Transdanubia region of Hungary. Using traditional qualitative and quantitative culture and molecular biological detection methods—complemented with microbiome analyses based on 16S rRNA sequencing—we aimed to provide additional evidence for the potential causative role of *Actinomyces* spp. in MRONJ.

## 2. Results

### 2.1. Characteristics of the MRONJ Patients and Control Subjects

The summary of the characteristics of MRONJ patients and controls is shown in Table 1. Samples corresponding to *n* = 35 MRONJ patients and *n* = 35 control subjects were analyzed during the study. The median age of control subjects was significantly lower (35 vs. 70 years; *p* < 0.001) compared to MRONJ patients, as they represented a cohort of otherwise healthy individuals, and the reason for their attendance at the Department was not related to a chronic condition. This was further underlined when observing the differences in the prevalence of DM (*p* < 0.001) and hypertension (*p* = 0.004) between the groups, because patients affected by these conditions all received medications for their corresponding co-morbidities. On the other hand, no significant differences were shown among the cases and controls in regard to the lifestyle factors (i.e., tobacco consumption, alcohol use) assessed in our study (*p* > 0.05 in all cases).

Among MRONJ patients, the majority of subjects were either Stage II (54.3%; *n* = 19) or Stage III (37.1%; *n* = 13). In total, 94.0% (*n* = 33) of subjects received antiresorptive medications due to an underlying malignant diagnosis and corresponding bone metastases. The majority of patients (91.0%; *n* = 32) received zoledronate intravenously (iv.), while one patient (3.0%) received iv. ibradronate, one patient (3.0%) received *per os* denosumab, and one patient (3.0%) received *per os* denosumab and *per os* ibradronate as their initial treatment, respectively. In the case of 74.3% (*n* = 26) patients, the switching of antiresorptive medications was necessary: these instances include changes from iv. zoledronate to *per os* ibadronate (*n* = 8 cases), from iv. zoledronate to iv. denosumab (*n* = 16 cases), and from iv. zoledronate to *per os* ibadronate and then to iv. denosumab (*n* = 2 cases). The median time elapsed between the initiation of antiresorptive treatment and the detection of the primary osteonecrotic lesion of the jawbones was 36 months (range: 8–162 months).

The surgical interventions that were performed are most commonly associated with the treatment of a pathological fracture, bone necrosis exceeding the alveolus, and the presence of an intra- or extra-oral fistula which causes constant complaints, or, in the case of maxillary involvement, the presence of an oroantral-, or oronasal fistula. Following the surgical interventions, all MRONJ patients (100.0%) required hospitalizations of various lengths (1–3 weeks), where intravenous antibiotic therapy was initiated, in line with national and international recommendations (amoxicillin–clavulanic was administered most often ± metronidazole. In the case of penicillin hypersensitivity [*n* = 1], clindamycin was prescribed). After discharge, *n* = 25 patients (71.4%) received sequential oral antibiotic therapy. Furthermore, alveolar correction, mandibular resection, and partial maxillary resection were also performed. Following the consultation with the patient’s treating physician—who ordered the antiresorptive treatments—a “drug holiday” (i.e., a periodic suspension of antiresorptive therapy) was initiated before any invasive interventions in the oral cavity for the majority of patients. Based on the follow-up on treated MRONJ patients, *n* = 0, *n* = 4, and *n* = 6 of patients had recurrences at 1 month, 3 months, and 6 months post-surgery, respectively (*n* = 10 overall; 28.6%). Recurrence was observed in the mandible in *n* = 7 patients and the maxilla in *n* = 3 patients, where either conservative treatment or an additional intervention was necessary. At 12 months post-surgery, *n* = 6 (17.1%) patients succumbed to their underlying illness.

The control group consisted of individuals who underwent tooth extraction or other corresponding interventions necessitating bone removal: in 74.3% (*n* = 26), the intervention was performed due to the removal of an impacted lower wisdom tooth; in these patients, the tooth removal was not performed by a traditional extraction, but often involved flap formation, the sectioning of the tooth, and bone removal. In the other 25.7% (*n* = 9) of cases, the reason for removal was focal debridement, the removal of teeth due to orthodontic treatment, or indications associated with prosthetic treatment.

### 2.2. Results of Microbiological Analyses: Traditional Qualitative and Quantitative Culture and PCR

Based on traditional aerobic and anaerobic culture methods, a mixed aerobic–anaerobic microbiota with a high colony count (expressed in CFU/mg) was cultivated from the bone samples of 34 out of 35 (97.1%) MRONJ patients; in the case of one patient, only a mixed aerobic flora was cultivated. The median number of different anaerobic species and aerobic species cultivated from patients was 5 (range: 0–14) and 1 (range: 0–4), respectively; fungal species were cultivated only in the case of two (6.0%) patients. In the control group, no cultivable anaerobic flora was found in 30.9% (*n* = 11) of samples. The median number of anaerobic species and aerobic species cultivated from patients was 2 (range: 0–6) and 1 (range: 0–3), respectively; fungal species were cultivated in the case of one (3.0%) patient. Overall, 185 different anaerobic strains, corresponding to 65 different species, were identified from the samples of MRONJ patients, while 72 anaerobic strains, corresponding to 27 different species, were identified from the samples of controls. The range, frequency, and distribution of isolated anaerobic species detected from cases and controls are summarized in Table 2. A considerably higher species richness may be observed in association with MRONJ patients.

Among the MRONJ patients, traditional quantitative culture after 12 days (detection limit ~10^4^ CFU/mg) strains were identified from one or more different *Actinomyces* spp. and/or ALOs in 65.8% (*n* = 23) of samples, while this rate was 17.1% (*n* = 6) in samples from controls (*p* < 0.001). In MRONJ patients, one *Actinomyces* spp. strain and/or ALOs was found in 9 cases, two different species were found in 13 cases, and isolates from three different species were found simultaneously in 1 case. The density of these bacteria of interest varied between 10^4^–10^9^ CFU/mg. In the controls, one isolate of *Actinomyces* spp. and/or ALOs was found in four cases, while isolates that belonged to two different species were found in one case; furthermore, the density of bacteria was lower, between 10^4^–10^5^ CFU/mg. The results of the quantitative culture, specific to *Actinomyces* spp. and/or ALOs in the tested samples, are shown in Table 3.

In addition to Actinomyces spp. and/or ALOs, the species of *Fusobacterium* spp., *Prevotella* spp., *Veillonella* spp., and GPAC were the most frequently isolated species in high colony counts. In comparison with the control group’s samples, *Fusobacterium* spp. (22 vs. 7; *p* = 0.001), *Prevotella* spp. (22 vs. 6; *p* = 0.034), and GPAC (30 vs. 9; *p* = 0.016) were significantly more common among MRONJ patient samples; on the other hand, no significant differences were shown for *Veillonella* spp. (30 vs. 25; *p* > 0.05) (Table 2). In both the MRONJ patient and control groups, the most common co-isolated aerobic bacteria were α-haemolytic, oral streptococci (*Streptococcus anginosus*, *S. constellatus*, *S. mitis*, *S. oralis*, and *S. sanguis*); surprisingly, in both groups, Gram-negative bacteria from the Enterobacterales order (*Escherichia coli*, *Klebsiella pneumoniae*, *Citrobacter* spp., and *Morganella morganii*), and strains belonging to *Acinetobacter* spp. were also among the isolated species.

In addition to traditional culture-based methods, bone samples were also subject to PCR analysis, using universal *Actinomyces*/ALO 16S ribosomal RNA (rRNA) gene primers; the results of the PCR assays for MRONJ and control samples are summarized in Table 3. The concordance between culture-positivity and PCR-positivity was 100.0% (23/23) in *Actinomyces*-positive MRONJ samples; furthermore, 6/6 samples (17.1%) were negative in both culture-based (after ≥12 days) and PCR assays. On the other hand, six (17.1%) culture-negative samples were positive in the PCR assays; overall, 82.9% (*n* = 29) of MRONJ samples were PCR-positive for *Actinomyces*/ALOs. No sample was noted as having a positive culture-based detection of *Actinomyces* while the 16S rRNA PCR was negative.

In the context of MRONJ disease stages, all samples from Stage I MRONJ patients were culture-negative, while 2/3 were PCR-positive for *Actinomyces*/ALOs. Among samples from Stage II patients, 14/19 samples were culture-positive, while the PCR assay detected one additional PCR-positive sample (15/19 overall). Finally, among Stage III patients, 9/12 samples were culture-positive, while the PCR-assay detected *Actinomyces*/ALOs in all but one (12/13) bone samples. Interestingly, *Actinomyces*/ALOs were detected from all (100.0%) patients’ samples who were affected by either HT or DM, respectively.

The concordance between culture-positivity and PCR-positivity was also 100.0% (6/6) in *Actinomyces*-positive samples from control subjects. Furthermore, among the 29 culture-negative samples, 16S rRNA PCR analysis has identified 7 additional PCR-positive samples; thus, PCR-positivity in control samples was 37.1% (*n* = 13) overall, similar to culture-positivity. The differences in PCR-positivity were significant between the MRONJ samples and samples from controls (82.9% vs. 37.1%; *p* < 0.001).

### 2.3. Microbiota Composition of Bone Samples from MRONJ Patients and Controls

As a part of our study, 5-5 patient samples (denoted as K1–K5 from controls and T1–T5 from MRONJ patients) were randomly selected before being aware of the results of traditional culture, and were subjected to 16S rRNA gene sequencing to highlight potential differences between the microbiota of the bone samples. The mean length of index 16S rRNA PCR products was 654 bp. Neither DNA isolation nor 16S rRNA PCR resulted in measurable amounts of DNA from the simultaneously processed transport buffers of samples as from the negative controls. A total of 2.88 million valid sequences were obtained, resulting in 2.1 million high-quality reads; the median number of reads within one sample was 212,000. Detailed information pertaining to the results of sequencing analyses, on a sample-by-sample basis, is shown in the Supplementary Material File S1.

**Figure 1:**
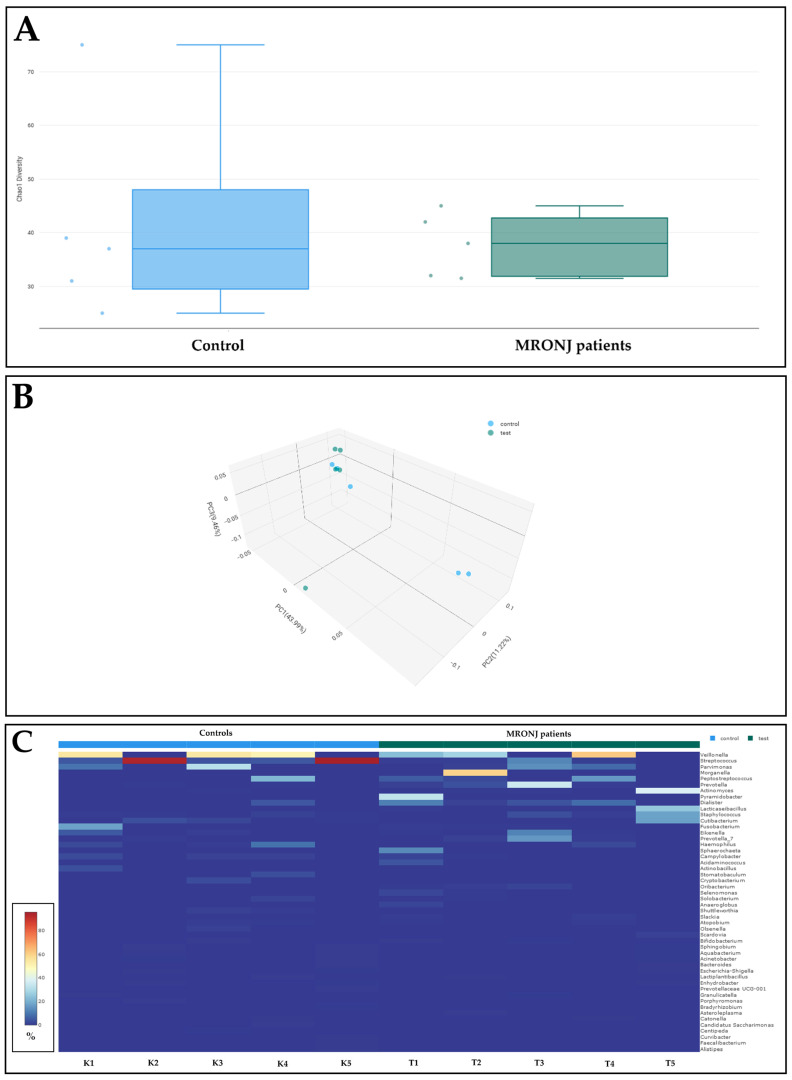
Comparison of Chao1 alpha diversity (**A**), Bray–Curtis PCoA beta diversity (**B**), and heatmap (**C**) of bacterial abundance (expressed as relative abundance%) in bone samples from controls (K1–K5) and MRONJ patients (T1–T5).

The results of the bacterial 16S rRNA gene sequencing are summarized in Figure 1., while the relative abundance (%) of the relevant bacteria taxa (i.e., ≥0.05% relative abundance) in bone samples from control (K) subjects and MRONJ patients (T) are shown in Table 4 and Table 5, respectively. When comparing Chao1 alpha diversity results corresponding to bone samples between control and MRONJ patients, no significant differences (*p* > 0.05) were found (Figure 1A). Furthermore, when comparing Bray–Curtis PCoA beta diversity results between control and MRONJ samples, no significant differences (*p* > 0.05) were observed (Figure 1B).

Among control samples, a considerable dominance of *Streptococcus* (especially in samples K2 and K5) and *Veillonella* (especially in K1, K3, and K4) was noted, while bacterial abundance rates were substantially more heterogeneous in MRONJ bone samples (Figure 1C, Table 4 and Table 5). Except for T5, where the dominance of *Actinomyces* in the bone microbiota was seen (relative abundance: 38.5%), notable differences were not observed among K (0–0.19%) and T1–T4 (0.05–0.46%) samples in the context of the relative abundance of *Actinomyces* (Table 4 and Table 5). The T5 sample corresponds to a patient with Stage III MRONJ, and long-term antiresorptive treatment due to prostate cancer and associated bone metastases. Of note, K1–K3 and K5 were samples that were negative for *Actinomyces*/ALOs both in the case of traditional culture and PCR-based assays; in contrast, T1, T2, and T5 samples were positive in both assays mentioned.

## 3. Discussion

MRONJ is a serious AE associated with the use of antiresorptive medications, leading to the necrosis of the bone tissue in the jaw; as limited possibilities are available for the management of this condition—especially in its advanced stages—it is a serious challenge for physicians in clinical care [9,10,11,12,13,14,28]. As the prevalence of MRONJ is steadily increasing worldwide, partly due to demographic changes and the emergence of more potent antiresorptive treatments, the precise knowledge of disease pathophysiology and the underlying patient-specific risk factors contributing to the development of the disease is essential to provide effective preventive and therapeutic interventions, given the preferences and expectations of patients [43]. The management of MRONJ may vary considerably according to disease stage and the attributes of the patients. However, as most of the population do not attend regular dental screening, many of the osteonecrotic lesions are only detected in their advanced stages [44]. Consequently, due to complications of the disease and the surgical resection of the jawbone, the QoL of patients may be considerably impaired. The evidence on the principal causes for the development of MRONJ is still controversial; however, numerous studies have explored the possible relationship between the composition of the oral microbiota and MRONJ, with studies using modern molecular biological methods and genomic sequencing providing scores of new information on the topic [45].

Oral bacteria may contribute to the pathomechanism of developing MRONJ via multifarious mechanisms. On the one hand, disruptions in the physiological balance of the oral microbiota—i.e., dysbiosis—may lead to a decrease in the species economy and a lower number and density of protective and anti-inflammatory microorganisms. Therefore, pathogenic bacteria, which cause inflammation and can contribute to bone death, may more readily proliferate [46]. Moreover, the oral microbiota plays a key role in the modulation of the host’s immune response; therefore, shifts in its composition—together with the effect of PPs—result in a compromised immune function, allowing pathogenic members of the oral microbiome to more easily colonize and infect tissues thus contributing to the development of MRONJ [47]. The presence of oral pathogens also promotes the production of inflammatory mediators (e.g., interleukins, chemokines) leading to a proinflammatory response, and they may also possess notable virulence factors (lipopolysaccharides, enzymes, and toxins) that lead to the breakdown of bone tissue [48]. Inflammatory mediators and microbial toxins may also lead to vascular damage and decreased microcirculation in the maxilla and/or mandible; this may contribute to the proliferation of other pathogenic anaerobic bacteria (due to the reduced supply of oxygen), and may adversely affect bone health [49]. Oral microorganisms tend to form mono- and multi-species biofilms on teeth and bone surfaces; the biofilm matrix forms a protective barrier against antibiotics and the cells of the immune system, and the acidic environment of the biofilm may also be a key element in the pathogenesis of MRONJ [50]. The next-generation sequencing-based study of Kim et al. highlighted a two-way relationship between the oral microbiota and the development of MRONJ lesions: Some species, like *Streptococcus*, *Lactobacillus*, *Bifidobacterium*, and other common members of oral microbiota with saccharolytic activity, create an anoxic, acidic microenvironment, which may further be enhanced by invasive procedures, trauma, dental infections, and antiresorptive treatment [45]. There have been numerous publications investigating the changes in the microbial composition of MRONJ lesions, but there is still no consensus among authors regarding the key bacterial species associated with MRONJ. As the possible role of *Actinomyces* spp. in the development and progression of MRONJ has been suggested by numerous publications, our present study sought to verify the presence of these bacteria in bone samples taken surgically from MRONJ patients from both a clinical and microbiological context, in comparison with healthy control subjects, using traditional culture-based and molecular biological techniques. To the best of our knowledge, this is the first similar study in Hungary, with such a comprehensive microbiological assessment, corresponding to MRONJ patients.

In our population (consisting of 35 MRONJ patients and 35 healthy controls), the culture-based positivity (performed in a traditional culture-based method in anaerobic atmosphere for at least 12 days) of bone samples for *Actinomyces*/ALOs was 65.8% and 17.1%, while the PCR-based detection rate was 82.9% and 37.1% from MRONJ and the control samples, respectively. These target species were significantly more commonly found in MRONJ samples in both cases, and samples from patients with osteonecrosis were characterized by considerably higher species richness (65 vs. 27 different species) as well. With the use of PCR, the recovery rate was improved by 25.9% in the case of MRONJ samples, and by 216.9% in the case of control samples, respectively, compared to the use of culture-based methods alone. The stage of the disease also had a considerable effect, as the rate of detection for *Actinomyces*/ALOs increased in more advanced stages of MRONJ. *Actinomyces*/ALOs were also commonly co-isolated with oral streptococci and *Fusobacterium* spp.; the synergistic interactions between these microorganisms have been extensively reported in the oral biofilm. However, *Fusobacterium* spp. may also have a synergistic role in the pathogenesis of the disease, which is supported by the results of animal experiments, showing that *F. nucleatum* infections in the extraction cavities of mice, following high-dose PP administration, result in delayed wound healing, leading to the exposure of the bone tissues [51]. The findings of Sedghizadeh et al. also support the notion that the multi-species microbial biofilm has a key role in the development of MRONJ. Their study assessed bone samples using traditional histopathological methods and scanning electron microscopy, where they have described the co-aggregation of *Actinomyces* species with specific genera, such as *Streptococcus*, *Fusobacterium*, *Bacillus*, *Staphylococcus*, *Selenomonas*, and *Treponema* [52].

Additionally, our results were complemented by 16S rRNA gene sequencing for a selection of bone samples in our study. Although in this context our results are only cross-sectional—and do not represent the entire population—they provide important insights into the microbiota of the MRONJ (T1–T5) and non-MRONJ (K1–K5) bone tissues. While no significant differences were identified (according to alpha and beta diversity measures), the contrast in the number of taxa represented clearly show that MRONJ bone samples maintained higher microbial diversity in their taxa; meanwhile, the control subject’s bone samples were consistently dominated by *Streptococcus* spp. and *Veillonella* spp. Also, there were no significant differences in the relative abundance of *Actinomyces* in the tested samples, whereas two extreme cases were identified: one control sample, where *Actinomyces* was not detected (0.0%), and one sample from a MRONJ-patient who belonged to Stage III, where its abundance was 38.50%. Modern metagenomic research has revealed new findings about MRONJ microbiota that suggest a close relationship between altered microbial diversity and the progression of the disease; these studies underscore the role of microbial colonization and the high prevalence of *Actinomyces* as a critical factor in the development of MRONJ [32,45,53,54].

*Actinomyces* spp. and related species are ubiquitous in the oral cavity among the early colonizing flora, but they may also cause chronic inflammatory processes; the hallmarks of these difficult-to-treat infections include granulomatous processes, the formation of fistulae, suppuration, and purulent discharge with a foul odor which are also common with the presentation of MRONJ. In the context of the detection and diagnosis of actinomycotic lesions, the histological examination and microscopy of a tissue sample, pus, or abscess is considered the gold standard, with a reported sensitivity of ~75.0% [36,55]. These samples often contain the characteristic “sulfur granules”, which are masses of filamentous bacteria, bound together by biofilm and inorganic components; however, these granules may be absent in 35–60% of cases [34,35,36,37,55,56]. Histological examination entails the use of various staining methods to enhance the successful detection of these bacteria: these may include Periodic acid Schiff (PAS), Gömöri’s meténamin, hematoxylin-eosin (HE), and fluorescent staining methods [57]. The Hungarian study of Brody et al. demonstrated the use of validated triple staining (Gram, PAS, and Grocott’s methenamine silver) on previously evaluated MRONJ histological samples—suspected for *Actinomyces*—using HE alone. The study has highlighted a considerable false negative rate through the use of traditional staining, as the re-evaluation has yielded 93.7% of positive samples (vs. 8.93%) [58]. Furthermore, the same authors noted the high false-negative rates of culture, as out of 39 samples with available culture results, only 2 (5.1%) were culture-positive [59]. The report of Kaplan et al., which was also based on the use of multiple staining methods to assess MRONJ-associated bone and other tissue samples, also corroborated these findings, highlighting that the rate of *Actinomyces*-associated lesions may not be as rare as previously reported. Furthermore, “*Actinomyces* density”, calculated by the authors, showed a significant positive correlation with the median length of antibiotic treatment [60]. A seemingly similar relationship was demonstrated by a recent study of Ibrahim et al., as they found that, among their patients with MRONJ or osteoradionecrosis, the condition of patients deteriorated rapidly where the presence of *Actinomyces* spp. was confirmed in their samples with a specific PCR assay [61].

The successful culture of *Actinomyces* largely depends on the sampling technique, and whether it was specifically searched for due to the clinician’s suspicion. Furthermore, considerable laboratory background and expertise are needed, due to the special culture requirements (anaerobic atmosphere, suitable culture media, incubation for at least 12 days) of these bacteria to be realized. Even so, the sensitivity of the culture is ~50.0%, and in addition to sampling and laboratory techniques, it may also be influenced by whether the patient previously received antibiotic therapy [34,35,36,37,55,56]. Nevertheless, this begs the question of whether the inconsistencies pertaining to the presence of *Actinomyces*/ALOs in MRONJ may partly be explained by the techniques used for their isolation and identification. In contrast, the rate of histological confirmation of *Actinomyces* from MRONJ tissue samples ranges from 12 to 100% [61,62,63]. The study of Russmueller et al. confirmed this, as out of the MRONJ-associated necrotic bone samples (taken from patients aged ≥65 years), 89.1% presented with *Actinomyces*-like filamentous rods [64]. Similarly, the retrospective study and literature review performed by Cerrato et al. (corresponding to the data of *n* = 114 patients overall) reported an 82.2% prevalence of *Actinomyces* from MRONJ patients’ samples [32]. As demonstrated by our results, as well as other studies published by other authors, the introduction of molecular biological techniques (PCR, 16S rRNA sequencing, whole-genome analysis) into routine clinical microbiology may yield better recovery rates for these species if they are specifically searched for, due to the greater sensitivity of these methods [65,66]. By applying the combination of histological examination and PCR, Hansen et al. found a similar positivity rate for *Actinomyces* spp. in their patients with clinical and radiological symptoms of MRONJ when compared to our results (20/31, 61.5%) [40].

As a part of our study, the underlying characteristics of the patients—corresponding to the development of MRONJ—were also described, as published studies overwhelmingly suggest that a multifactorial etiology supports the development of the disease. The age group represented in our sample corresponds well to the age when the condition is most commonly detected [67]. Nevertheless, a slight female dominance has also been reported previously [15,16,17,18], which was not confirmed in our case, although this may be explained by the study’s sample size. The overwhelming majority (94.0%) of patients were affected by malignancy, and most patients had some chronic underlying conditions. Otherwise, the lifestyle factors were not considerably different between MRONJ patients and control subjects. MRONJ more commonly affects the mandibular bone, which also concurs with our experience [68]. The median time elapsed from the initiation of antiresorptive treatment and diagnosis of MRONJ was 36 months, which is in line with previous reports, highlighting that the second–third year of therapy is where the onset of symptoms is most commonly seen [69].

While existing evidence confirms that the characteristics (indication, type, dose, and mode of administration) of the antiresorptive treatment prescribed and the underlying characteristics of the patient are definitely significant, the results from our current study show that alterations in the oral microbiome—with *Actinomyces* and related species being the most relevant—play a key role in the development of MRONJ, which without a doubt, interact with other factors described. These results strengthen the “infection hypothesis” for the development of MRONJ, where—through facilitated access due to PP treatment—infections resulting from the colonization of tissues by *Actinomyces* lead to chronic inflammation that contributes to osteomyelitis and then the necrosis of bone tissue in the jaw [31,33,34,35,36,37,38,39,40,45,50,51,52,53,54,55,64,70]. As the causal pathophysiological mechanisms of the MRONJ–microbiome axis are still unknown, further investigations are needed to determine the exact role of the presence of *Actinomyces* in osteonecrosis of the jaw.

However, it must be noted that there are a substantial number of studies providing convincing results that challenge the thesis of the “infection hypothesis” and the oral microbiome’s direct role in causing MRONJ. For example, the role and dysfunctions of the immune system (“immunological hypothesis”) in MRONJ are increasing; on the one hand, through their pharmacological activity, PPs may directly impair the function of the immune system—leading to increased susceptibility to oral infections—the mechanisms of which were summarized in detail by Roato et al. [71]. The co-morbidity with systemic immunological diseases, such as rheumatoid arthritis and DM, may affect the resilience of the immune system and the body’s ability to respond to infection and inflammation [72]. It has been suggested that patients with MRONJ have reduced immune resistance, which may affect their ability to cope with immunological stress caused by PP treatment [73]. Moreover, results obtained through the field of pharmacogenetics may also carry considerable weight, due to the role of specific SNPs for *FDPS*, *SIRT1*, *CYP2C8*, and the VEGF receptor, and the increased risk of MRONJ [21,22,74]. However, the assessment of these genetic determinants in our patients was outside the scope of the present study.

Our study possesses several limitations, which should be taken into consideration when interpreting our findings: (i) the sample size of patients/bone samples assessed in the study, while providing valuable insights, were achieved through a convenience sampling approach, and may not be representative of the diversity of all microbiome compositions that could contribute to MRONJ, affecting the external validity of our findings; (ii) the age of MRONJ patients and the presence of chronic underlying conditions shows considerable variation when compared to the healthy control group—which is partly a consequence of the inclusion/exclusion criteria set—introducing bias to our study. Therefore, it is unknown whether these underlying diseases or malignancies affected the bone microbiota composition; (iii) while no significant differences were reported between the two groups in the context of the lifestyle factors studied, it must also be noted that these data were self-reported; therefore, a more comprehensive analysis (e.g., patient’s diet, habits, environmental factors) is needed to ascertain their potential effects. To address points (ii) and (iii), a case–control study design would be needed. Furthermore, (iv) only a random selection of 5-5 samples were subjected to 16S rRNA sequencing analyses, which additionally affects the external validity of our study. To complement, verify, and extend our cross-sectional insights achieved by 16S rRNA gene sequencing, the involvement of additional samples from more patients (containing individuals from all different stages of MRONJ), preferably within a more robust, multicentric study design, and their comparison with age and gender-matched controls would be desirable. Nonetheless, as our study employed well-defined and sound laboratory methodologies, the internal validity of our study is ensured in its specific context. Our study has demonstrated—using a wide variety of microbiological methods—that *Actinomyces* is more commonly found in the necrotic bone tissue of patients receiving antiresorptive drugs, compared to the samples of non-affected controls, indicating that they may play a role in the clinical course and prognosis of MRONJ. While the current study cannot ascertain the precise role of the oral microbiota—and more specifically *Actinomyces* species—in MRONJ disease progression (i.e., whether an opportunistic pathological process or benign colonization occurs), the relative abundance of these species in patients (over healthly controls) increased, which may carry key therapeutic implications. As current evidence suggests a multifactorial etiology for the development of the disease, future studies may examine the functional differences in the microbiome by means of metagenomic and metaproteomic analyses to show which proteins are overexpressed during potentially pathological processes. Furthermore, changes in the diversity of the oral microbiota and potential synergistic interactions—in relation to metabolomics, or otherwise—between *Actinomyces* and other anaerobic species of interest may be a hallmark of the development of MRONJ. Finally, in a more holistic approach, the study of the interactions between these opportunistic pathogens and the host immune system is also warranted. The detection of *Actinomyces* may be carried out via traditional culture and molecular biological methods, but histology remains the mainstay for diagnosis: the confirmation of the presence of *Actinomyces* in the relevant samples necessitates attention, as it should inform therapeutic strategies, and may alter the patient’s prognosis. Physicians also need to be made aware of recent taxonomic reclassifications affecting the *Actinomyces* genus [37]. In the clinic, the principal aim is to prevent the occurrence of MRONJ, considering the most important risk factors, and by being diligent with the choices related to antiresorptive pharmacotherapy, as the treatment of infections caused by *Actinomyces* requires long-term antibiotic therapy combined with surgical debridement, with the success of treatment (including conservative and surgical methods) being unpredictable. Furthermore, *Actinomyces* spp. isolates with increased minimum inhibitory concentrations and phenotypic resistance against various beta-lactam antibiotics have also been described [56].

## 4. Materials and Methods

### 4.1. Study Design and Setting, Inclusion and Exclusion Criteria

The present study was carried out at the Department of Dentistry, Oral and Maxillofacial Surgery, University of Pécs (PTE) Clinical Centre between 1 June 2023 and 31 January 2024. The Clinical Centre is a 1725-bed tertiary care university teaching hospital situated in the Southern Transdanubia region of Hungary, providing primary to tertiary healthcare services to ~800,000 inhabitants, according to NHIF data [75,76]. During the study, participants belonged to two groups: the MRONJ patients’ group and a control group. The MRONJ patients’ group consisted of patients who presented at the Department of Dentistry, Oral and Maxillofacial Surgery to undergo surgery, who met the following criteria: (i) the patients had MRONJ-related complaints, visible denuded bone surfaces, and osteonecrotic lesions (i.e., MRONJ stages I.–IV.) in the oral cavity, where the use of only conservative therapy could not be used in the long term in view of the complaints, clinical symptoms, and the advanced state of the process (at-risk patients and patients in MRONJ stage 0 were not eligible, as denuded, necrotic bone surfaces are not yet visible; therefore, sampling would not have been possible) [11,12], (ii) the patients had received *per os* or iv. PPs, or other antiresorptive medications, either due to malignant or non-malignant causes.

Conversely, the control group consisted of otherwise healthy, non-pregnant adults undergoing tooth extraction (corresponding to wisdom teeth or other teeth) or other corresponding interventions necessitating bone removal at the Department, who met the following criteria: (i) no history of osteoporosis, malignant disease (or chemotherapy), hereditary or acquired immunosuppression, (ii) no history of osteomyelitis or other forms of jaw osteonecrosis, (iii) no history of receiving antiresorptive, antiangiogenic medications that may cause osteonecrosis, or radiotherapy of the head–neck region, (iv) no antibiotic therapy ≥2 months before intervention. Overall, *n* = 35 individuals were involved in the MRONJ patients’ group and the control group, respectively.

In addition to bone sampling for qualitative and quantitative microbiological analyses, data were collected on the participants’ comorbidities, lifestyle factors, the state of oral hygiene, characteristics and reasons for antiresorptive treatment, and the nature of the presenting lesions. MRONJ patients were further classified as low- or high-risk, based on MRONJ risk factors [11,12]. In the oral cavity, clinical and imaging methods (panoramic X-rays and cone beam computer tomography [CBCT] examinations) were used to examine the type of possible restorations, missing teeth, and the extent of the denuded necrosis. The status and conditions of MRONJ patients were followed up on during a maximum of a 12-month period post-surgery.

### 4.2. Bone Sampling Procedure

Bone sampling was carried out during surgery, both in the case of the MRONJ patients’ group and the control group, respectively. Five minutes before the surgical intervention or tooth-removing procedures, the affected area was washed for 1–2 min intra-orally, and the entire oral cavity was disinfected with a 0.2% chlorhexidine solution. In the case of MRONJ patients, the removal of the necrotic bone was with a curative aim, where the extent and technique used during the operative intervention were based on the level of the necrosis and corresponding clinical recommendations. A piece (approximately 2–3 mg in weight) of necrotic bone removed from the patient via a sterile surgical tool (e.g., bone forceps, drill)—which was a good representation of the histological characteristics of the necrosis—was placed in anaerobic transport medium (Copan MSwab^®^, Copan, Bresica, Italy) with sterile forceps. In the case of five randomly selected patients, part of the bone sample was frozen directly after sampling to allow for sequencing analyses. Bone sampling for the study did not involve additional, repeated interventions, pain, or stress (physical and psychological) for the patients, as the sampling was performed from a necrotic bone piece that would have been removed anyway under local anesthesia or narcosis. The sample was taken before the initiation of antibiotic therapy.

Within the same period, sampling was carried out for the control group, with alveolar bone fragments (approximately 0.5–1 cm in size)—removed during the extraction of wisdom teeth, other teeth, or tooth roots out of necessity during the surgical procedure, either due to the anatomical location of the tooth or due to the technique—similarly placed in anaerobic transport medium (Copan MSwab^®^); the study did not involve additional interventions to harvest bone fragments, other than those necessitated by the control’s dental treatment. Similarly to MRONJ patients, in the case of five randomly selected controls, part of the bone sample was frozen directly after sampling to allow for sequencing analyses.

Before the microbiological processing of the obtained samples, the weight of all bone pieces was measured to allow for the determination of colony-forming units/milligrams (CFU/mg) in subsequent analyses.

### 4.3. Microbiological Processing of Samples, Traditional Qualitative and Quantitative Culture Methods

The obtained bone samples (i.e., the necrotic bone pieces in the MRONJ patients’ group and the alveolar bone pieces from the controls) were processed immediately after arrival at the Department of Medical Microbiology and Immunology, University of Pécs; the time elapsed between bone sampling and processing were <30 min in all cases. The bone pieces received were placed in 1.0 mL of reduced Brain Heart Infusion (BHI; pH 7.2) broth (Oxoid, Basingstoke, UK), and then homogenized for 30 s (using Stomacher 80, Labsystem, Budapest, Hungary). A series of dilutions were prepared from the stock solution, which was diluted to 10^−1^–10^−6^. In total, 100–100 µL of the stock solution and each of the serial dilutions were plated parallelly on selective and non-selective agar media. To isolate and count all culturable anaerobic bacteria, Schaedler agar (containing horse blood 5% *v*/*v*, haemin, and Vitamin K_1_; bioMérieux, Marcy l’Etoile, France) supplemented with 5% (*v*/*v*) bovine blood, haemin, and Vitamin K_1_ was used [77]. Columbia blood and chocolate agar media (bioMérieux, Marcy l’Etoile, France) were used for the isolation and count of aerobic/microaerophilic bacterial flora. Endo medium (bioMérieux, Marcy l’Etoile, France) was used for the selective cultivation of potentially occurring aerobic Gram-negative species of the Enterobacterales order, while Sabouroud Dextrose Agar (SDA; bioMérieux, Marcy l’Etoile, France) medium was used for the cultivation of fungi. Cultures on the solid agar plates were incubated for 5 days in an anaerobic chamber (Bactron Sheldon Man, Cornelius, OR, USA), with an anaerobic atmosphere (90% N_2_, 5% H_2,_ and 5% CO_2_) or a CO_2_ atmosphere, respectively (depending on the agar), for 48 h at 37 °C. Bone fragment samples were incubated in Fastidious Anaerobe Broth (FAB; bioMérieux, Marcy l’Etoile, France) for another 12 days and then repeatedly cultured on solid media [56,77]. The cultured bacteria and fungi were counted by determining the exact count of colonies, and the strains with different colony morphology were identified at the species level using matrix-assisted laser desorption–ionization time-of-flight mass spectrometry (MALDI-TOF MS; Bruker Daltonics, Bremen, Germany). To perform bacterial identification using the MALDI-TOF assay, bacterial cells with different colony morphologies were transferred to a stainless-steel target, where an on-target extraction was performed by adding 1 µL of 70% formic acid prior to the matrix. Following drying at room temperature, the cells were covered with 1 µL of matrix (α-cyano-4-hydroxy cinnamic acid in 50% acetonitrile/2.5% trifluoro-acetic acid; Bruker Daltonics, Bremen, Germany) [77]. The microFlex LT Biotyper instrument (Bruker Daltonics, Bremen, Germany; in a positive linear mode across the m/z range from 2 to 20 kDa; for each spectrum, 240 laser shots at 60 Hz in groups of 40 shots per sampling area were collected) was used for mass spectrometry analysis. For the analysis of spectra, the MALDI Biotyper RTC 3.1 software (Bruker Daltonics, Bremen, Germany) and the MALDI Biotyper Library 3.1 were used. After analysis, a log(score) value was assigned to all isolates, indicating the reliability of MALDI–TOF MS identification [77]. Based on consensus criteria, the genus-level identification of bacteria isolates was deemed reliable if the log(score) after MALDI measurements was ≥1.7, while for reliable species-level identification, a log(score) ≥2.0 was needed [78]. The results of the quantitative culture were in terms of CFU/mg.

### 4.4. DNA Extraction and PCR Amplification

The starting samples for DNA extraction comprised of 1.5 mL of anaerobic cultures with the bone sample grown in anaerobic BHI broth medium for five days at 37 °C. Total genomic DNA was extracted from the centrifuged pellet from the 1.5 mL of anaerobic culture without dilution, with the E.Z.N.A^®^ Bacterial DNA kit (D3350-00, Omega Bio-tek, Norcross, GA, USA) following a short protocol optimized for difficult to lyse microorganisms, as recommended by the manufacturer. This method included a pre-incubation step with 10 μL of lysozyme solution for 2 h at 37 °C, and mechanical lysis with 25 mg of Glass Beads S for 5 min to disrupt the cells before samples were digested with proteinase K at 55 °C for an hour. Then, the bacterial lysate was treated with RNAse A solution for 5 min at room temperature to eliminate microbial RNA. After isolation on a HiBind column (Omega Bio-Tek, Norcoss, GA, USA), the concentration and purity of the extracted DNA were determined with a NanoDrop One/One Microvolume UV-VIS spectrophotometer (Thermo Fisher Scientific, Waltham, MA, USA).

During the qualitative, end-point PCR assay, the universal primers AMY-16S-F (5′-GGCKTGCGGTGGGTACGGGC-3′) and AMY-16S-R (5′-GGCTTTAAGGGATTCGCTCCRCCTCAC-3′), previously published by Xia et al. [79], were used to amplify the 16S rRNA gene region of the order *Actinomycetales*, which includes the genus *Actinomyces* and related species. The PCR reactions were conducted in a total of 25 μL volume on a thermal cycler (T100, Bio-Rad Laboratories, Inc. Hercules, CA, USA). The reaction mix contained 1 μL of template DNA, 2.5 μL of 10× DreamTaq^TM^ Green buffer, 0.5 μL of 10 mM dNTPs, 0.5 μL of each primer (10 μM), 0.25 μL of DreamTaq^TM^ Green DNA Polymerase (5 U/μL), and 19.75 μL of nuclease-free water. PCR was carried out using the following cycle parameters: initial denaturation at 95 °C for 3 min, 35 cycles of denaturation at 95 °C for 30 s, annealing at 68 °C for 30 s, elongation at 72 °C for 1 min, and a final extension at 72 °C for 10 min. The final PCR products were evaluated by 1% agarose gel electrophoresis and visualized under ultraviolet (UV) light using the Molecular Imager Gel Doc^TM^ XR+ system (Bio-Rad Laboratories, Inc. Hercules, CA. USA). The images were analyzed by the Image Lab^TM^ v6.0.1 build 34 software (Bio-Rad Laboratories Inc., Hercules, CA, USA). Amplicon size was analyzed by comparison to a GenRuler 100 bp DNA ladder (Thermo Fisher Scientific, Waltham, MA, USA). Isolated DNA from the type strain of A. *oralis* (VPI 12593/CDC W1544/) and a five-day aerobic culture of *Enterobacter cloaceae* complex in BHI broth was used as *Actinomycetales*-PCR positive (+) and negative (–) controls, respectively [80]. After analysis, the amplified PCR products were stored at −20 °C until further processing.

### 4.5. DNA Isolation, 16S rRNA Gene Library Preparation Protocol and MiSeq Sequencing

Out of the *n* = 35 MRONJ patients and *n* = 35 controls, 5-5 patient samples (denoted as K1–K5 and T1–T5 in the following) were randomly selected, where 16S rRNA gene sequencing was performed to provide cross-sectional insights and to compare the differences in the microbiome of the two groups of subjects. DNA isolation from the MRONJ patients’ and contols’ bone samples was performed using the ZymoBIOMICS DNA Miniprep Kit (Zymo Research Corp., Irvine, CA, USA). The DNA concentration of the samples was measured using a Qubit fluorimeter with a Qubit dsDNA HS Assay Kit (Thermo Fisher Scientific, Waltham, MA, USA). Bacterial DNA was amplified with labeled primers covering the V3-V4 region of the bacterial 16S rRNA gene. PCR and DNA purifications were performed according to the Illumina protocol [81]. PCR product libraries were evaluated using a DNA 1000 kit and an Agilent 2100 Bioanalyzer (Agilent Technologies, Waldbronn, Germany). The equimolar concentrations of the libraries were pooled, and next-generation sequencing (NGS) was performed using MiSeq^®^ Reagent Kit vs3 (600 cycles PE) in an Illumina MiSeq System (Illumina, San Diego, CA, USA). Extraction-negative controls and PCR-negative controls were included in every run. Raw sequencing data were retrieved from Illumina BaseSpace, and data were analyzed using the CosmosID [82] bioinformatics platform. The CosmosID-HUB Microbiome’s 16S workflow for taxonomy and species-level identification was conducted with DADA2’s naive Bayesian classifier using the Silva version 138 database.

### 4.6. Statistical Analysis

During the data analysis of the participants’ characteristics, continuous variables were expressed as a median and range (minimum–maximum), while categorical variables were expressed as frequencies and percentages (*n*, %). Normality testing was carried out using the graphical method (Q-Q diagrams) and Shapiro–Wilk tests. The χ^2^-test and Fisher’s exact tests were used to determine differences between proportions, while Mann–Whitney U-tests were used for the comparison of continuous variables between cases and controls. The abovementioned statistical analyses were performed using SPSS Statistics version 22.0 (IBM Inc., Chicago, IL, USA).

Statistically significant differences between bacterial taxa abundances measured in K and T samples were calculated via the Mann–Whitney U test. Statistical significance between cohorts was implemented via the Wilcoxon rank sum test for Chao1 alpha diversity and the permutational multivariate analysis of variance (PERMANOVA) analysis for Bray–Curtis principal coordinate analysis (PCoA) beta diversity using the statistical analysis support application of CosmosID [83]. In addition, the CosmosID bioinformatics platform was used for LEfSe (linear discriminant analysis effect size) to identify distinctive microbial features between sample groups (CosmosID Inc., Germantown, MD, USA). During both groups of analyses, *p* values < 0.05 were considered statistically significant.

### 4.7. Ethical Considerations

The study was conducted in accordance with the Declaration of Helsinki and national and institutional ethical standards. Ethical approval for the study protocol was obtained from the Human Institutional and Regional Biomedical Research Ethics Committee, Medical School, University of Pécs (9503-2023 University of Pécs (PTE)/2023).

## Figures and Tables

**Table 1:** Socio-demographic characteristics and anamnestic data of MRONJ patients and controls.

	MRONJ Patients (*n* = 35)		Controls (*n* = 35)		*p*-Value
**Age**					
Median [years] (minimum–maximum)	70 (40–87)		35 (17–78)		*p* < 0.001 (Mann-Whtiney U test)
	***n***	**%**	***n***	**%**	
**Sex**					
Male	16	45.7	18	51.4	n.s. (χ^2^-test)
Female	19	54.3	17	48.6	
**Co-morbidities**					
Hypertension (HT)	24	68.5	5	14.3	*p* = 0.004 (Fisher’s exact test)
Diabetes mellitus (DM)	11	30.9	3	8.6	*p* < 0.001 (Fisher’s exact test)
HT and DM	10	28.6	0	0	*p* < 0.001 (Fisher’s exact test)
Obesity (BMI ≥ 30)	1	3.0	0	0	n.s. (Fisher’s exact test)
**Lifestyle factors**					
Tobacco consumption (T)	12	34.3	19	54.3	n.s. (χ^2^-test)
Alcohol consumption (A)	6	17.1	9	25.7	n.s. (Fisher’s exact test)
T and A	5	14.3	3	8.6	n.s. (Fisher’s exact test)
Illicit substance use	0	0	0	0	n.s. (Fisher’s exact test)
**Indication for antiresorptive treatment**					
Prostate cancer	14	39.9	n.r.		
Breast cancer	11	30.9			
Lung cancer	3	8.6			
Kidney cancer	3	8.6			
Colon cancer	1	3.0			
Myeloma multiplex (MM)	1	3.0			
Osteoporosis	1	3.0			
Langerhans-hystiocytosis	1	3.0			
**MRONJ staging**					
Stage I.	3	8.6	n.r.		
Stage II.	19	54.3			
Stage III.	13	37.1			
**Localization of primary lesion**					
Maxilla	12	34.3	n.r.		
Mandible	22	62.8			
Both jawbones	1	2.9			

BMI: body mass index; MRONJ: medication-related osteonecrosis of the jaws; n.r.: not relevant; n.s.: not significant; Stage I–III. in accordance with the AAOMS classification.

**Table 2:** Frequency and distribution of anaerobic isolates from traditional anaerobic culture of samples from MRONJ patients and controls.

	MRONJ Patients Number of Isolates (*n* = 185 overall)	Controls Number of Isolates (*n* = 72 overall)
Gram-positive anaerobic rods	***n* = 54 overall**	***n* = 25 overall**
*Abiotrophia adiacens*	1	0
*A. defectiva*	1	1
***Actinotignum schaalii***	**2**	**0**
***A. naeslundii***	**10**	**2**
***A. oralis***	**2**	**0**
***A. oris***	**5**	**4**
***A. viscous***	**1**	**0**
***Gleimella europaeus***	**1**	**0**
***Schaalia meyeri***	**1**	**0**
***S. odontolytica***	**13**	**2**
***S. turicensis***	**1**	**0**
*Bifidobacterium dentium*	2	0
*B. longum*	1	0
*Clostridium beijernickii*	2	0
*Cutibacterium acnes*	0	2
*C. avidum*	2	0
*C. namnetense*	0	1
*Eggerthella lenta*	0	4
*Eubacterium brachy*	1	0
*E. cataniformis*	1	0
*E. cellulosolvens*	1	0
*Filifactor alocis*	1	1
*Lactobacillus paracasei*	1	0
*Lachroanaerobaculum orale*	0	7
*Lacticaseibacterium spp.*	0	1
*Paraclostridium bifermentans*	1	0
*Solobacterium moorei*	3	0
Gram-positive anaerobic cocci	***n* = 30 overall**	***n* = 9 overall**
*Gemella haemolisans (morbillorum)*	2	0
*G. sanguis*	0	1
*Lactococcus lactis*	1	0
*Lancefieldella rimae*	2	0
*Oslenella uli*	1	0
*Parvimonas micra*	11	5
*Peptoniphilus lacrimalis*	1	0
*P. stomatis*	8	3
*Peptoniphilus spp.*	2	0
*Peptostreptococcus anaerobius*	2	0
Gram-negative anaerobic rods	***n* = 71 overall**	***n* = 15 overall**
*Aggregatibacterium aphrophilus*	1	1
*Bacteroides fragilis*	2	0
*Campylobacter rectus*	2	0
*Capnocytophaga sputigena*	2	0
*Dialister microaerophilus*	1	0
*Eikenella corrodens*	6	0
*Fusobacterium canaliformis*	2	0
*F. mortiferum*	1	0
*F. necrophorum*	3	0
*F. nucleatum*	15	6
*F. periodonticum*	0	1
*F. varium*	1	0
*Leptotrichia hofstadii*	1	0
*L. wadei*	2	1
*L. trevisani*	1	0
*Porphyromonas gingivalis*	5	0
*P. somerae*	1	0
*Prevotella baroniae*	1	0
*P. buccae*	5	2
*P. buccalis*	1	0
*P. dentalis*	1	0
*P. denticola*	5	2
*P. heparinolytica*	1	0
*P. intermedia*	2	1
*P. jejuni*	1	0
*P. melaninogenica*	1	0
*P. nigrescens*	3	0
*P. oralis*	1	1
*Selenomonas artemidis*	1	0
*S. sputigena*	1	0
*Slackia exigua*	1	0
Gram-negative anaerobic cocci	***n* = 30 overall**	***n* = 25 overall**
*Veillonella atypica*	8	3
*V. dispar*	2	1
*V. parvula*	16	18
*V. rogosae*	4	3

MRONJ: medication-related osteonecrosis of the jaw; *Actinomyces* spp. and ALOs are denoted in ***boldface***.

**Table 3:** Results of quantitative culture and *Actinomyces*/ALO-specific PCR assays corresponding to bone samples from MRONJ patients and controls.

Sample ID	Culture Results *Actinomyces* or ALO Species Recovered		Colony Forming Units (CFU/mg)		Results of PCR Analyses	
	MRONJ Cases	Controls	MRONJ Cases	Controls	MRONJ Cases	Controls
1	*A. naeslundii*	*A. naeslundii*	10^9^	10^4^	+	+
2	*S. odontolytica*	negative	10^6^	0	+	+
3	*A. schaalii*	negative	10^9^	0	+	+
4	*A. naeslundii* *S. odontolytica* *A. oralis*	negative	10^9^ 10^6^ 10^5^	0	+	−
5	negative	negative	0	0	+	−
6	negative	negative	0	0	+	−
7	*A. oralis*	negative	10^4^	0	+	−
8	*S. odontolytica* *A. oralis*	negative	10^9^	0	+	+
9	*S. odontolytica* *A. oris*	*A. naeslundii*	10^9^	10^5^	+	−
10	*S. odontolytica*	*S. odontolytica* *A. oris*	10^9^	10^5^	+	−
11	negative	negative	0	0	−	−
12	*A. naeslundii* *S. odontolytica*	negative	10^9^	0	+	+
13	negative	negative	0	0	−	−
14	*S. odontolytica* *A. oris*	*A. oris*	10^9^	10^4^	+	+
15	negative	negative	0	0	+	−
16	*S. odontolytica* *G. europaeus*	*A. oris*	10^9^	10^5^	+	+
17	*A. naeslundii* *S. odontolytica*	negative	10^9^	0	+	−
18	*A. naeslundii*	negative	10^5^	0	+	−
19	*S. odontolytica* *S. meyeri*	negative	10^9^	0	+	+
20	*A. naeslundii* *A. oris*	negative	10^9^	0	+	−
21	negative	negative	0	0	−	−
22	*S. odontolytica*	negative	10^8^	0	+	−
23	*A. schaalii* *A. naeslundii*	*S. odontolytica* *A. oris*	10^7^	10^4^	+	+
24	*A. naeslundii* *S. odontolytica*	negative	10^5^	0	+	+
25	*S. odontolytica* *A. oris*	negative	10^5^	0	+	−
26	negative	negative	0	0	+	−
27	negative	negative	0	0	+	+
28	*A. naeslundii* *A. oris*	negative	10^7^	0	+	−
29	*S. turicensis*	negative	10^5^	0	+	−
30	*A. naeslundii* *A. viscous*	negative	10^5^	0	+	−
31	negative	negative	0	0	−	+
32	negative	negative	0	0	+	−
33	*A. oris*	negative	10^4^	0	+	−
34	negative	negative	0	0	−	−
35	negative	negative	0	0	−	−

ALO: *Actinomyces*-like organism; CFU: colony-forming unit; MRONJ: medication-related osteonecrosis of the jaw; PCR: polymerase chain reaction; −: PCR-negative; +: PCR-positive.

**Table 4:** Relative abundance of relevant bacteria taxa in bone samples from control (K) subjects.

Samples				
K1	K2	K3	K4	K5
*Veillonella*: 55.10%	*Streptococcus*: 93.89%	*Veillonella*: 54.67%	*Veillonella*: 50.40%	*Streptococcus*: 95.4%
*Fusobacterium*: 17.41%	*Cutibacterium*: 3.35%	*Parvimonas*: 30.33%	*Peptostreptococcus*: 21.64%	*Bacteroides*: 0.35%
*Parvimonas*: 9.07%	*Prevotella*: 0.30%	*Streptococcus*: 3.83%	*Haemophilus*: 8.88%	*Aquabacterium*: 0.33%
*Eikenella*: 4.65%	*Sphingobium*: 0.27%	*Cryptobacterium*: 2.98%	*Streptococcus*: 4.71%	*Prevotella*: 0.30%
*Actinobacillus*: 3.26%	*Porphyromonas*: 0.27%	*Cutibacterium*: 1.91%	*Dialister*: 4.69%	*Sphingobium*: 0.29%
*Campylocbacter*: 2.73%	*Parvimonas*: 0.22%	*Olsenella*: 1.79%	*Stomatobaculum*: 3.28%	*Acinetobacter*: 0.25%
*Haemophilus*: 2.23%	*Acinetobacter*: 0.20%	*Campylobacter*: 0.98%	*Solobacterium*: 1.75%	*Escherichia-Shigella*: 0.22%
*Staphylococcus*: 0.37%	*Murdochiella*: 0.14%	*Shuttleworthia*: 0.56%	*Campylocbacter*: 1.22%	*Bradyrhizobium*: 0.20%
*Granulicatella*: 0.11%	*Aquabacterium*: 0.12%	*Eikenella*: 0.44%	*Staphylococcus*: 1.15%	*Enhydrobacter*: 0.12%
*Aggregatibacter*: 0.06%	*Bacteroides*: 0.11%	*Bifidobacterium*: 0.44%	*Lactiplantibacillus*: 0.41%	*Cutibacterium*: 0.11%
*Bacteroides*: 0.05%	*Eschierichia-Shigella*: 0.11%	*Haemophilus*: 0.28%	*Shuttleworthia*: 0.31%	*Faecalibacterium*: 0.09%
***Actinomyces*: 0.05%**		*Atopoboium*: 0.28%	*Saccharimonas*: 0.24%	*Curvibacter*: 0.08%
		*Centipeda*: 0.20%	*Atopobium*: 0.19%	*Staphylococcus*: 0.08%
		***Actinomyces*: 0.19%**	*Prevotella*: 0.13%	*Parabacteroides*: 0.08%
		*Bacteroides*: 0.05%	*Cartonella*: 0.10%	*Allistipes*: 0.07%
			*Alloscardovia*: 0.09%	*Blautia*: 0.06%
			*Parvimonas*: 0.08%	*Atopostipes*: 0.06%
			*Fusobacterium*: 0.07%	*Agarthobacter*: 0.05%
			*Sphingobium*: 0.05%	*Finegoldia*: 0.05%
			***Actinomyces*: 0.05%**	*Veillonella*: 0.05%
				*Parvimonas*: 0.05%
				***(Actinomyces*: 0.01%)**

Only taxa with ≥0.05% relative abundance in the samples were included in the table. *Actinomyces* is highlighted in colored cells and denoted in **boldface**.

**Table 5:** Relative abundance of relevant bacteria taxa in bone samples from MRONJ (T) subjects.

Samples				
T1	T2	T3	T4	T5
*Pyramidobacter*: 32.35%	*Morganella*: 57.38%	*Prevotella*: 34.71%	*Veillonella*: 62.67%	***Actinomyces*: 38.50%**
*Veillonella*: 25.22%	*Veillonella*: 30.12%	*Prevotella_7*: 16.86%	*Peptostreptococcus*: 13.39%	*Lacticaseibacillus*: 24.39%
*Sphaerochaeta*: 12.71%	*Prevotella*: 3.53%	*Parvimonas*: 13.82%	*Dialister*: 7.92%	*Cutibacterium*: 17.17%
*Dialister*: 11.36%	*Peptostreptococcus*: 2.36%	*Streptococcus*: 12.70%	*Parvimonas*: 7.48%	*Staphylococcus*: 16.81%
*Peptostreptococcus*: 5.15%	*Dialister*: 1.25%	*Eikenella*: 11.50%	*Haemophilus*: 2.20%	*Scardovia*: 1.05%
*Acidaminococcus*: 4.20%	*Prevotella_7*: 1.12%	*Dialister*: 4.07%	*Slackia*: 0.85%	*Sphingobium*: 0.20%
*Selenomonas*: 2.22%	*Parvimonas*: 1.08%	*Staphylococcus*: 3.19%	*Streptococcus*: 0.73%	*Aquabacterium*: 0.20%
*Anaeroglobulus*: 1.76%	*Streptococcus*: 0.82%	*Oribacterium*: 1.58%	*Staphylococcus*: 0.65%	*Acinetobacter*: 0.15%
*Campylobacter*: 1.51%	*Oribacterium*: 0.51%	***Actinomyces*: 0.46%**	*Prevotella*: 0.60%	*Bacteroides*: 0.13%
*Prevotella*: 0.98%	*Asteroleplasma*: 0.29%	*Granulicatella*: 0.19%	*Atopobium*: 0.40%	*Escherichia-Shigella*: 0.09%
*Streptococcus*: 0.57%	***Actinomyces*: 0.20%**	*Bidifobacterium*: 0.17%	*Prevotella_7*: 0.025%	*Enhydrobacter*: 0.06%
*Fusobacterium*: 0.55%		*Gemella*: 0.15%	*Eikenella*: 0.13%	
*Lacticaseibacillus* 0.38%			*Catonella*: 0.09%	
*Bifidobacterium* 0.35%			*Neisseria*: 0.09%	
*Slackia*: 0.33%			*Rothia*: 0.06%	
*Oribacterium*: 0.18%			***Actinomyces*: 0.06%**	
*Prevotella_7*: 0.16%				
*Treponema*: 0.12%				
*Eikenella*: 0.10%				
*Parvimonas*: 0.10%				
*Bacteroides*: 0.07%				
***Actinomyces*: 0.05%**				
*Phascolarctobacterium*: 0.05%				

Only taxa with ≥0.05% relative abundance in the samples were included in the table. *Actinomyces* is highlighted in colored cells and denoted in **boldface**.

## Data Availability

The datasets generated and analyzed during the current study are available in the Short Read Archive (SRA) under the accession number PRJNA1221230/www.ncbi.nlm.nih.gov (accessed on 8 of February 2025).

## Supplementary Materials

The following supporting information can be downloaded at https://www.mdpi.com/article/10.3390/antibiotics14020203/s1: File S1: Detailed information pertaining to the results of sequencing analyses on a sample-by-sample basis.
